# Conserved and highly expressed tRNA derived fragments in zebrafish

**DOI:** 10.1186/s12867-015-0050-8

**Published:** 2015-12-22

**Authors:** Ana Raquel Soares, Noémia Fernandes, Marisa Reverendo, Hugo Rafael Araújo, José Luís Oliveira, Gabriela M. R. Moura, Manuel A. S. Santos

**Affiliations:** Department of Medical Sciences and Institute for Biomedicine–iBiMED, University of Aveiro, 3810-193 Aveiro, Portugal; IEETA, University of Aveiro, 3810-193 Aveiro, Portugal

**Keywords:** tRNA derived fragments, Zebrafish, Small non coding RNAs, tRNAs

## Abstract

**Background:**

Small non-coding RNAs (sncRNAs) are a class of transcripts implicated in several eukaryotic regulatory mechanisms, namely gene silencing and chromatin regulation. Despite significant progress in their identification by next generation sequencing (NGS) we are still far from understanding their full diversity and functional repertoire.

**Results:**

Here we report the identification of tRNA derived fragments (tRFs) by NGS of the sncRNA fraction of zebrafish. The tRFs identified are 18–30 nt long, are derived from specific 5′ and 3′ processing of mature tRNAs and are differentially expressed during development and in differentiated tissues, suggesting that they are likely produced by specific processing rather than random degradation of tRNAs. We further show that a highly expressed tRF (5′tRF-Pro^CGG^) is cleaved in vitro by Dicer and has silencing ability, indicating that it can enter the RNAi pathway. A computational analysis of zebrafish tRFs shows that they are conserved among vertebrates and mining of publicly available datasets reveals that some 5′tRFs are differentially expressed in disease conditions, namely during infection and colorectal cancer.

**Conclusions:**

tRFs constitute a class of conserved regulatory RNAs in vertebrates and may be involved in mechanisms of genome regulation and in some diseases.

**Electronic supplementary material:**

The online version of this article (doi:10.1186/s12867-015-0050-8) contains supplementary material, which is available to authorized users.

## Background

Small non-coding regulatory RNAs (sncRNAs) play fundamental roles in many aspects of biology and their classification is based on their size, structure, biogenesis and function [1]. MicroRNAs (miRNAs) constitute the most extensively studied class of sncRNAs and are known to regulate the expression of target genes at the translational level [2]. The advent of next generation sequencing (NGS) allowed for the identification and production of miRNA profiles [3–6], identification of piRNAs [7], 21-U RNAs [8] and rasiRNAs [9]. More recently, sncRNAs up to 30 nucleotides derived from tRNAs—the tRNA derived fragments (tRFs)—have also been identified [10, 11]. Fragments of tRNA have been retrieved by many computational analysis of NGS datasets, but they were initially considered random degradation products of tRNAs and were discarded from further analysis [12]. However, recent experimental data showed that they are a stable and functional class of sncRNAs produced by specific cleavage of certain tRNAs [10, 11, 13–15], and can be classified according to their origin, namely 5′tRFs, also known as tRF-5 series, which derive from 5′ processing of the mature tRNAs; 3′tRFs, also known as tRF-3 series, which derive from 3′ processing of the mature tRNA and 3′U-tRFs, or tRF-1 series, which derive from pre-tRNA processing by RNAse Z cleavage [11, 16]. Different studies have also demonstrated that some 5′- and 3′tRFs are generated by Dicer cleavage, similarly to miRNAs [10, 17], and that some of them are incorporated into Argonaute (Ago) proteins [13, 18] or are associated with piwi proteins [15]. Indeed, deep sequencing of HeLa cells identified 5′tRFs generated by Dicer cleavage at the D-loop, both in vitro and in vivo [10]. Dicer-dependent generation of 3′tRFs also occurs in a human embryonic kidney 293 cell line and luciferase reporter assays demonstrated that they silence target genes, suggesting that they are involved in gene regulation [18]. Moreover, a Dicer dependent 3′tRF is down-regulated in B cell lymphoma and represses the RPA1 gene, which is involved in DNA repair, similarly to miRNAs [17]. This functional similarity between tRFs and miRNAs may happen because some tRNAs have the potential to form a long hairpin as an alternative to the typical tRNA cloverleaf secondary structure, thus functioning as a Dicer substrate, as is the case of the tRNA^Ile^ in mouse embryonic stem cells [19].

Besides tRFs, longer tRNA fragments (30–38 nt) corresponding to tRNA halves are also produced by cleavage at the 5′ or 3′ sides of the tRNA anticodon by specific endonucleases, rather than Dicer, in response to nutritional deprivation and other stress conditions [20, 21], as is the case in *Tetrahymena thermophila* during amino acid starvation [22]. The 5′ derived tRNA halves induced by stress (tiRNAs) in human cells have the ability to inhibit translation initiation [23] and trigger the formation of stress granules [24], due to a terminal oligoguanine motif [25], indicating that these molecules are involved in gene expression regulation.

We have previously applied NGS to the discovery of zebrafish sncRNAs and identified novel miRNAs in this organism [26]. A new analysis of the sequencing datasets described herein identified 10 new tRFs that originate from specific cleavage of tRNAs. Expression analysis by northern blot shows that these tRFs are differentially expressed at different developmental stages and in certain tissues and their abundance is higher than the corresponding mature tRNA. Our data show that a 5′tRF, namely 5′tRF-Pro^CGG^ can be generated by Dicer and has trans-silencing ability, indicating that it may enter the RNAi pathway, controlling gene expression of specific targets. Northern blot and computational analysis also demonstrate that those tRFs are conserved in vertebrates and are differentially expressed in some disease states, namely during infection and cancer, suggesting their involvement in the mechanisms underlying diseases and their potential use as disease biomarkers.

## Results

### Identification of tRFs in zebrafish

**Fig. 1:**
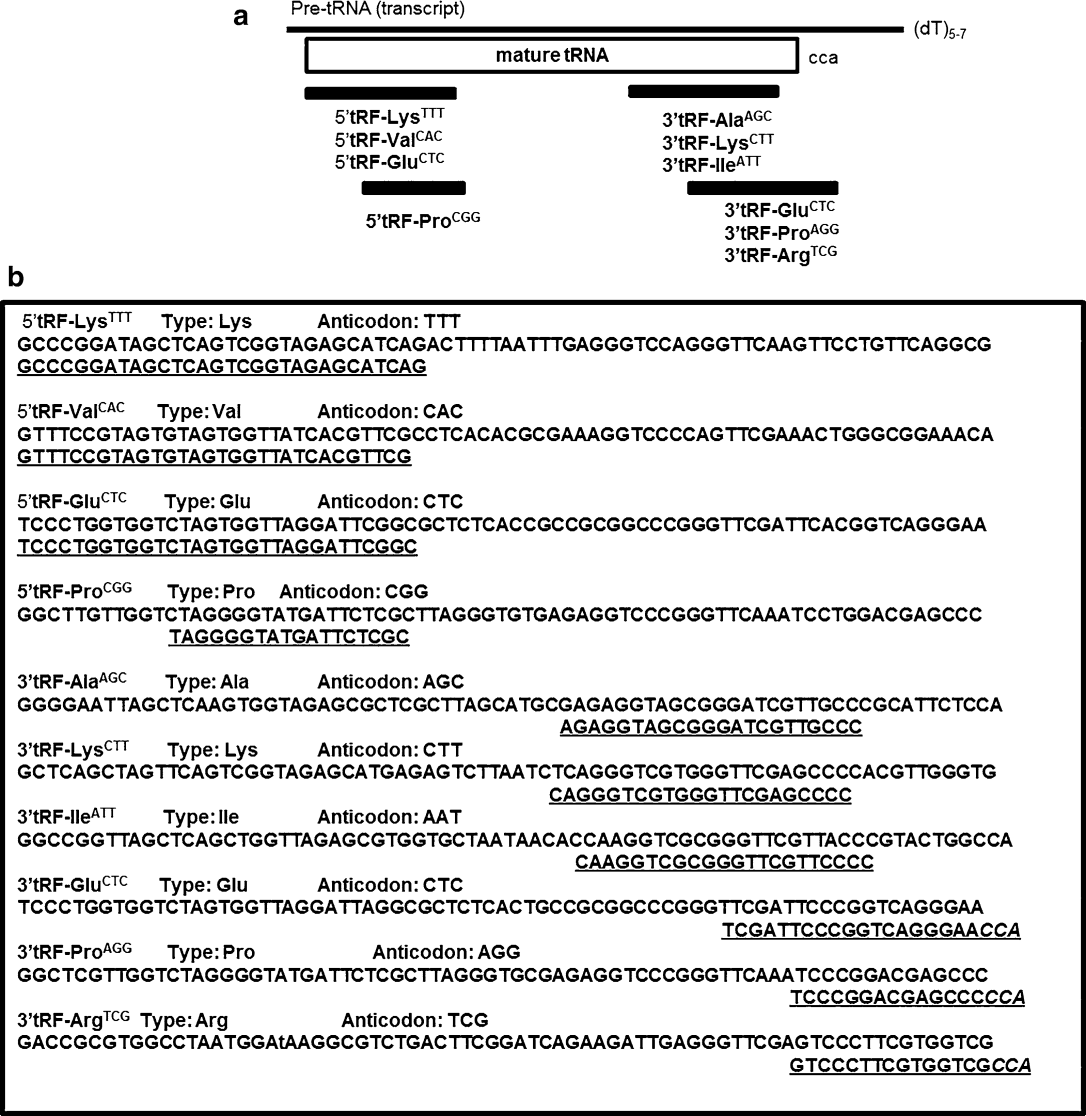
Classification of the zebrafish tRNAfs identified by deep sequencing. **a** Schematic view of the localization of the tRFs in the linear structure of mature tRNAs. Six tRFs align in the 3′ end of the mature tRNAs and 4 tRFs align to the 5′ region of the tRNA. **b** Alignment of the 12 tRFs in the corresponding mature tRNA

The Roche 454 NGS platform (max nr reads = 100,000) was used previously by our group to identify miRNA molecules in zebrafish adult tissues and developmental stages [26]. In order to identify sequences corresponding to other non-coding RNAs besides miRNAs, the retrieved reads were aligned against a database of known small RNAs extracted from Biomart/Ensembl, including snRNAs, snoRNAs, rRNAs and tRNAs, as described in the “Methods” (Additional file 1: Figure S1). 8 % of total sequencing reads matched the selected ncRNAs, where 61 % of them matched known tRNA loci. The majority of these reads matched to one particular structural domain of tRNAs, suggesting specific processing rather than random tRNA degradation, as described previously [10, 12]. We have considered specific cleavage whenever a given tRF sequence appeared more than three times in the cDNA libraries and the overall tRF alignments with a mature tRNA were dominated by that specific tRF. This methodology identified a total of ten tRFs, which aligned with the 3′ end of tRNAs (six 3′tRFs) and with the 5′end (four 5′tRFs) (Table 1; Fig. 1).

**Table 1:** Zebrafish tRFs identified by deep sequencing

tRF ID	Sequence (5′–3′)	Nr of reads	Expression
5′tRF-Lys^TTT^	GCCCGGATAGCTCAGTCGGTAGAGCATCAG	4	Adult tissues
5′tRF-Val^CAC^	GTTTCCGTAGTGTAGTGGTTATCACGTTCG	4	Adult tissues
5′tRF-Glu^CTC^	TCCCTGGTGGTCTAGTGGTTAGGATTCGGC	26	Development, adult tissues
5′tRF-Pro^CGG^	TAGGGGTATGATTCTCGC	12	Development, adult tissues
3′tRF-Ala^AGC^	AGAGGTAGCGGGATCGTTGCCC	16	Adult tissues
3′tRF-Lys^CTT^	CAGGGTCGTGGGTTCGAGCCCC	4	Adult tissues
3′tRF-Ile^AAT^	CAAGGTCGCGGGTTCGTTCCCC	6	Adult tissues
3′tRF-Glu^CTC^	TCGATTCCCGGTCAGGGAACCA	24	Development, adult tissues
3′tRF-Pro^AGG^	TCCCGGACGAGCCCCCA	13	Development, adult tissues
3′tRF-Arg^TCG^	GTCCCTTCGTGGTCGCCA	7	Adult tissues

tRF identification, sequence, number of reads and expression are shown

The tRFs identified in this study were 18–30 nt long and half of the 3′tRFs had the trinucleotide signature CCA at the 3′end, which was similar to previous reported cases [10, 11].

In zebrafish 12292 genes code for the different tRNAs, according to the genomic tRNA database. Those with highest copy numbers are: Lysine (1478 genes), Glycine (1162 genes), Leucine (1132 genes) and Serine (1065 genes). Although the 10 tRFs identified derived from different classes of tRNAs, there seems to be no correlation between the number of tRNA genes and the generation of tRFs, as most of these abundant tRNAs did not generate tRFs, with exception of Lysine (Fig. 1b). There was a slight enrichment in tRFs generated from Lysine, Glutamine and Proline tRNAs (Fig. 1b), suggesting that the generation of tRFs was not dependent on tRNA expression levels. 5′tRF-Lys^TTT^ and 3′tRF-Lys^CTT^ derived from different tRNA^Lys^ isoacceptors, 5′tRF-Glu^CTC^ and 3′tRF-Glu^CTC^ derived from tRNA^Glu^, whereas 5′tRF-Pro^CGG^ and 3′tRF-Pro^AGG^ derived from tRNA^Pro^ isoacceptors.

### tRF profiling

In order to confirm the deep sequencing data, the expression patterns of four tRFs, namely 5′tRF-Lys^TTT^, 5′tRF-Glu^CTC^, 5′tRF-Pro^CGG^ and 3′tRF-Pro^AGG^, were studied in different developmental stages [24 h post fertilization (hpf), 48, 72 and 96 hpf] and in different zebrafish adult tissues (brain, fins, bone/muscle, skin) using northern blot analysis, as described in the “Methods”.

**Fig. 2:**
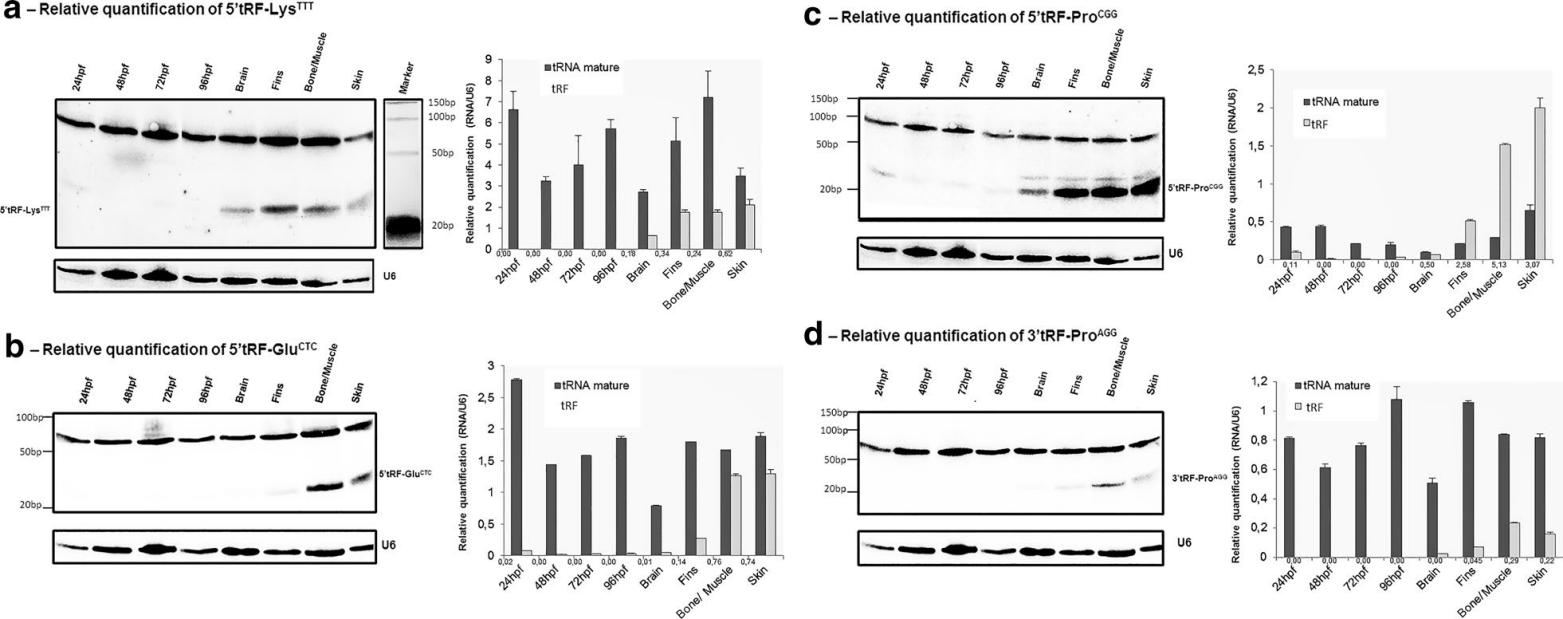
Quantification of four different tRFs by northern blot analysis. Total RNA was extracted from samples corresponding to different zebrafish developmental stages [24 h post fertilization (hpf), 48, 72, 96 hpf] and from samples of different zebrafish adult tissues, namely brain, fins, muscle and skin. 20 μg of total RNA from each sample was electrophoresed on 10 % PAA gels and transferred onto Hybond-N membranes for northern blot analysis. tRFs showed high hybridization signal in muscle and skin samples and low hybridization signal in developmental samples. U6 RNA was used as an internal positive control. Relative quantification of the bands corresponding to mature tRNAs and tRFs was carried out using U6 RNA as reference for normalization. Membranes were stripped and reprobed (membrane one was used to perform the following northern blots: 5′tRF- Lys^TTT^, 5′tRF-Pro^CGG^ and U6; membrane two was used to perform the following northern blots: 5′tRF-Glu^CTC^, 3′tRF-Pro^AGG^ and U6). Ratio between tRF and mature tRNA are indicated *under the bars* of each sample. **a** 5′tRF-Lys^TTT^ is expressed in adult tissues only. **b** 5′tRF-Glu^CTC^ is highly expressed in muscle and skin tissues. At 24 hpf, the level of mature tRNA is almost twofold higher than in the other samples. **c** 5′tRF-Pro^CGG^ is more abundant than the mature tRNA in fins, muscle and skin tissues. The expression of this fragment in skin is twofold higher than the mature tRNA. **d** 3′tRF-Pro^AGG^ is expressed at low level and is found in adult tissues only. *Data* are presented as the mean ± SD (n = 3)

There was poor correlation between the abundance of tRFs and mature tRNAs (Fig. 2a–d). During zebrafish development (from 24 to 96 hpf), only 5′tRF-Glu^CTC^ and 5′tRF-Pro^CGG^ were detected and at low levels (less than 0.2 tRF/U6 relative expression). These tRFs were expressed at high levels in bone/muscle and skin (>1 tRF/U6 relative expression), regardless of the levels of mature tRNAs in those tissues (Fig. 2b, c). Remarkably, the abundance of 5′tRF-Pro^CGG^ was higher than that of its corresponding mature tRNA in most tissue samples (fins, bone/muscle and skin, with a tRF/tRNA ratio of 2.58, 5.13 and 3.07, respectively), suggesting that it may have a functional role (Fig. 2c).

The other tRFs tested were not detected by northern blotting during development (Fig. 2a, d), but were detected in adult tissues. The abundance of the 5′tRF-Lys^TTT^ was similar in fins, muscle and skin (~1.5 tRF/U6 relative expression), whereas the abundance of the corresponding mature tRNA was higher in muscle (7 tRNA/U6 relative expression) than in other tissues (Fig. 2a). 3′tRF-Pro^AGG^ was also detected in adult tissues only, at low levels—maximum 0.3 tRF/U6 relative expression (Fig. 2d).

The lack of correlation between the mature tRNAs and the respective tRFs abundance in different tissues suggested that tRF generation is a regulated process, rather than a random degradation process. Moreover, almost no bands of intermediate molecular weight were detected in the northern blots, indicating that tRNA cleavage occurs at specific cleavage sites. In some tissues, namely brain, fins and skin a band of intermediate size ~30 nt was detected for 5′tRF-Pro^CGG^ (Fig. 2c), however the smaller band, corresponding to 5′tRF-Pro^CGG^ was always the most prominent one, indicating preferential accumulation of this tRF.

**Fig. 3:**
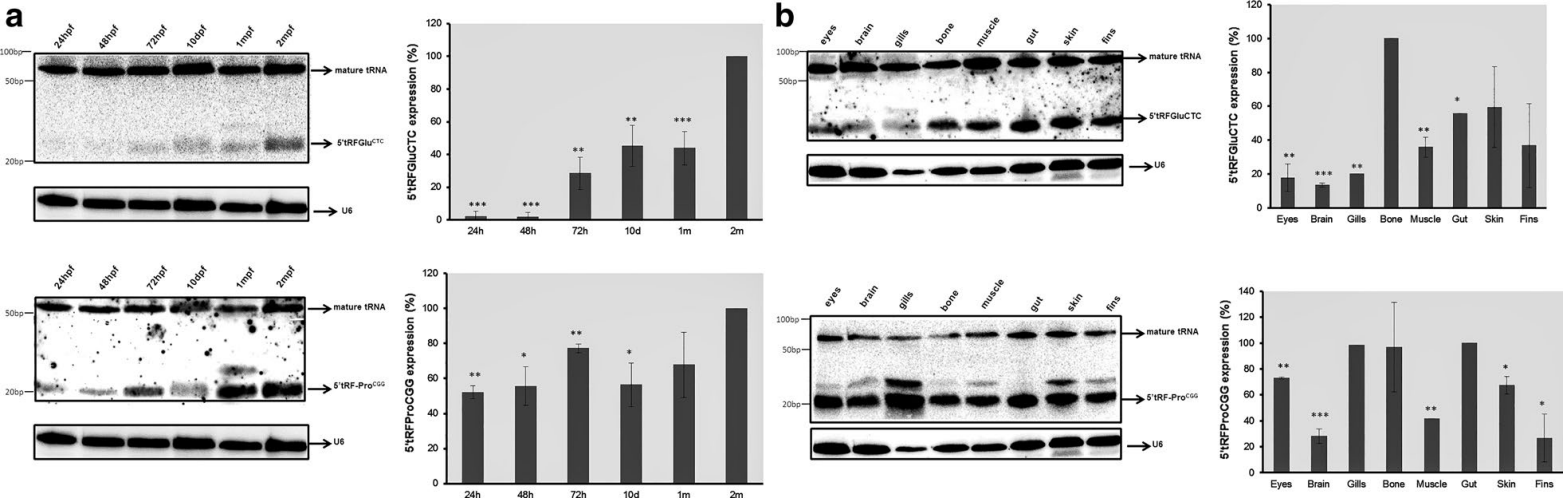
Embryo and adult profiling of 5′tRF-Glu^CTC^ and 5′tRF-Pro^CGG^. 20 μg of total RNA was fractionated in a 10 % PAA and transferred onto Hybond-N membranes for northern blot analysis. U6 RNA was used as an internal positive control. Relative quantification of the bands corresponding to tRFs was carried out using U6 RNA as reference for normalization. The relative expression level (%) was calculated considering that the sample with the highest level of tRF expression corresponded to 100 % of tRF abundance. **a** Northern blot analysis of 5′tRF-Glu^CTC^ and 5′tRF-Pro^CGG^ in different developmental stages, namely 24, 48, 72 hpf, 10 days post fertilization (dpf), 1 month post fertilization (mpf) and 2 mpf. Membrane was stripped and probed with 5′tRF-Glu^CTC^, 5′tRF-Pro^CGG^ and U6. The highest level of expression was detected at 2 mpf for both tRFs. 5′tRF-Glu^CTC^ expression gradually increased during development. 5′tRF-Pro^CGG^ was already detected at 24 hpf and its expression varied slightly through development. **b** Northern blot analysis of 5′tRF-Glu^CTC^ and 5′tRF-Pro^CGG^ using samples from different differentiated tissues, namely eyes, brain, gills, bone, muscle, gut, skin and fins. Membrane was stripped and probed with 5′tRF-Glu^CTC^, 5′tRF-Pro^CGG^ and U6. 5′tRF-Glu^CTC^ was highly expressed in bone, gut, skin and fins and less expressed in brain. The highest level of 5′tRF-Pro^CGG^ expression was detected in gills, bone and gut. This tRF was expressed at lower levels in the brain, similarly to 5′tRF-Glu^CTC^. Data are presented as the mean ± SD (n = 3) (Student’s unpaired *t* test, *P < 0.05, **P < 0.01, ***P < 0.001)

Since northern blot analysis revealed that both 5′tRF-Glu^CTC^ and 5′tRF-Pro^CGG^ were highly abundant tRFs, we have focused our attention on these two molecules and studied them in more detail. We have extended our previous developmental analysis up to 2 months post fertilization (mpf) (Fig. 3a). The levels of both 5′tRF-Pro^CGG^ and 5′tRF-Glu^CTC^ were highest at 2 mpf and for this reason the tRF expression was considered 100 % at this particular stage. 5′tRF-Glu^CTC^ was barely detected at 24 and 48 hpf, but its levels increased steadily during development (Fig. 3a). 5′tRF-Pro^CGG^ was detected already at 24 hpf and there was an increased generation of this tRF at 72 hpf (30 % increase), however at 10 days post fertilization (dpf) the 5′tRF-Pro^CGG^ levels decreased to levels similar to those observed at 24 and 48 hpf and increased again reaching high abundance at 2 mpf.

These tRFs are also differentially produced in a variety of tissues (Fig. 3b). The expression of both tRFs in bone is approximately twofold higher than in muscle. In fact, the levels of the 5′tRF-Glu^CTC^ were highest in bone and for comparative purposes its relative expression was considered 100 % in this tissue. Its relative expression was also high in skin (60 %) and gut (58 %) and lower in eyes (18 %), brain (15 %) and gills (20 %). This data confirmed the initial profiling data which showed that 5′tRF-Glu^CTC^ generation was low in brain and high in skin (Fig. 2). The 5′tRF-Pro^CGG^ showed the maximal abundance in gills and for this reason we considered its relative expression as 100 % in this sample. The relative abundance of this tRF was also variable between tissues: eyes (75 %), bone (98 %), skin (70 %) and gut (98 %) and lower in brain and fins (<30 %), confirming the initial profiling data (Figs. 2c, 3b). Therefore, different tRFs are differentially produced and accumulated in the various tissues of zebrafish, in particular both 5′tRF-Glu^CTC^ and 5′tRF-Pro^CGG^ are generated at lower levels in the brain than in any other tissues tested.

### Biogenesis of tRFs

**Fig. 4:**
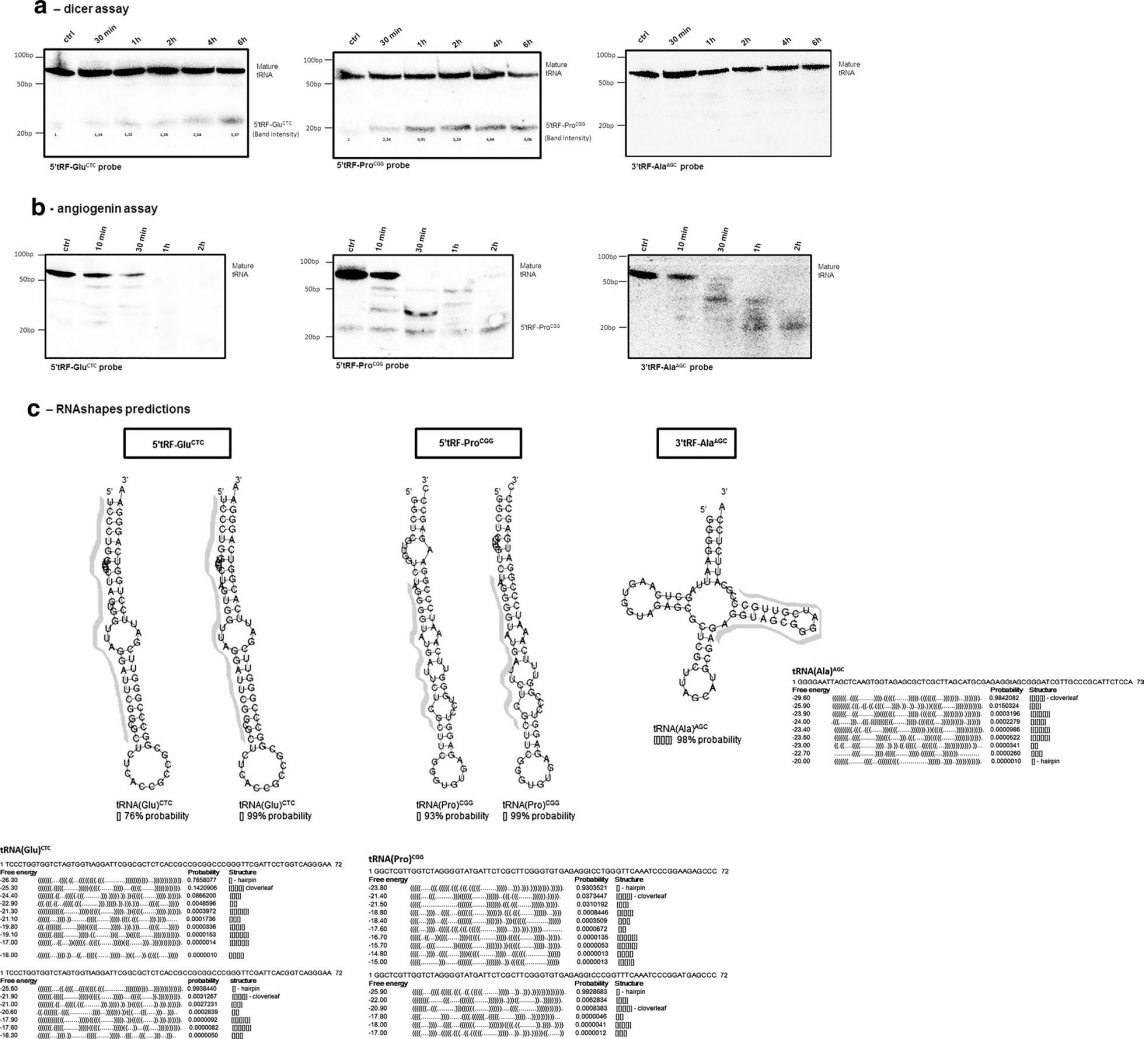
Biogenesis of tRFs. 20 µg of total RNA were incubated with 2 U of recombinant Dicer or 1 µM of Angiogenin and was fractionated on 10 % PAGE, transferred to Hybond-N membranes and subjected to northern blot analysis. **a** 5′tRF-Glu^CTC^ and 5′tRF-Pro^CGG^ are produced by Dicer in vitro and its abundance increased over time, suggesting that the corresponding mature tRNAs are efficient Dicer substrates. Band intensity values normalized for the total RNA used are depicted for the tRFs generated over time by Dicer. For each tRF, the control RNA sample (first *blot lane*) was incubated without the enzyme for 6 h at 37 °C. The 3′tRFAla^AGC^ probe hybridized with the mature tRNA only and did not detect any tRF after Dicer incubation. **b** Analysis of 5′tRF-Glu^CTC^ and 5′tRF-Pro^CGG^ and 3′tRFAla^AGC^ production by Angiogenin showed unspecific cleavage of the mature tRNA, suggesting that Angiogenin was not involved in the biogenesis of any of the tRFs tested. **c** RNA shapes prediction of alternative structures of the mature tRNAs that originated 5′tRF-Glu^CTC^ and 5′tRF-Pro^CGG^. The probabilities of formation of pre-miRNA hairpin-like structures (represented by []) is shown. The higher (>90 %) alternative folding probabilities of the 5′tRF-Pro^CGG^ precursors [tRNA(Pro)] is consistent with the higher cleavage efficiency of this tRNA, as shown in the *northern blots* in **a**. The tRNA(Ala)^AGC^ does not originate tRFs after Dicer cleavage and its transcript folds as a typical mature tRNA. Sequences of tRFs of designed northern blot probes are highlighted in *grey*. The results of RNA shapes for each tRNA are shown below the secondary structures. The free energy, the probability of folding and the folding structures are depicted

Previous studies have implicated Dicer (RNAse III family member) in tRF generation [10, 18]. To test whether Dicer could be responsible for 5′tRF-Glu^CTC^ and 5′tRF-Pro^CGG^ biogenesis we incubated total RNA from 72 hpf zebrafish embryos with this enzyme and performed northern blot analysis. Both tRFs were detected after 30 min of incubation with Dicer and their abundance increased over time (Fig. 4a), suggesting that this enzyme was involved in tRNA^Glu^ and tRNA^Pro^ cleavage and in the biogenesis of 5′tRF-Glu^CTC^ and 5′tRF-Pro^CGG^, respectively. Dicer did not produce non-specific tRNA cleavage products, suggesting that this enzyme is directly involved in the biogenesis of both 5′tRF-Glu^CTC^ and 5′tRF-Pro^CGG^ in vitro. As Dicer has also been implicated in the generation of 3′tRFs [17, 18], we tested its ability to generate 3′tRF-Ala^AGC^, however, we have only observed bands corresponding to the mature tRNA(Ala)^AGC^, indicating that Dicer is not involved in the generation of this particular 3′tRF in vitro (Fig. 4a).

Angiogenin, an RNAseA family member has also been implicated in the generation of some tRNA derived fragments, namely tRNA halves, in response to stress [20, 23, 24]. Another study also shows that angiogenin is involved in the production of 20–25 nt tRFs in vitro in HEK293 cells [27]. Our experiments confirmed that angiogenin is not involved in the generation of the tRFs tested, (Fig. 4b), reinforcing the role of Dicer in the biogenesis of 5′tRF-Glu^CTC^ or 5′tRF-Pro^CGG^ and indicating that 3′tRF-Ala^AGC^ generation is probably dependent on the action of alternative ribonucleases. Besides we did not detect any increase in 5′tRF-Glu^CTC^ or 5′tRF-Pro^CGG^ production upon exposure to stress (data not shown), but the tRNAs were cleaved into short non-specific tRNA fragments after 10 min of incubation with Angiogenin (Fig. 4b). Longer incubation times increased the degradation of tRNA and after 2 h the complete fraction of mature tRNA was degraded. Moreover, angiogenin degraded completely the fragments of the tRNA(Glu), but was not able to degrade the 5′tRF-Pro^CGG^, indicating that these tRFs are not angiogenin targets (Fig. 4b).

Recent computational analysis also suggested that Dicer cleavage of tRNAs may occur when the primary transcripts form long hairpins, as an alternative fold to the standard tRNA cloverleaf secondary structure [19]. To test this hypothesis, we determined the probability of the tRNA transcripts forming pre-miRNA like folds using RNAshapes [28]. The Shape Probability option was used to calculate the shape probabilities based on the partition function where the probability of a shape is the sum of the probabilities of all structures that fall into this shape. 3′tRF-Ala^AGC^ can only derive from one tRNA(Ala) locus, while 5′tRF-Glu^CTC^ and 5′tRF-Pro^CGG^ can be assigned to different tRNA(Glu) and tRNA(Pro) locus in zebrafish (>100 tRNA locus), as found in the genomic tRNA database [29]. We have tested the folding of all of them using RNAShapes and verified that 5 % of tRNA(Glu) and 50 % of tRNA(Pro) transcripts are more prone to acquire a hairpin like folding than any other folding. In most of these cases, the probability of hairpin-like folding is higher than 70 %. Figure 4c shows some of the examples of alternative folds of tRNA(Glu) and tRNA(Pro) obtained, supporting the hypothesis that 5′tRF-Glu^CTC^ and 5′tRF-Pro^CGG^ can be produced by Dicer. On the other hand the mature tRNA(Ala)^AGC^ showed 98 % probability of forming a typical cloverleaf tRNA-like structure (Fig. 4c).

### 5′tRF-Pro^CGG^ has the ability to silence gene expression

**Fig. 5:**
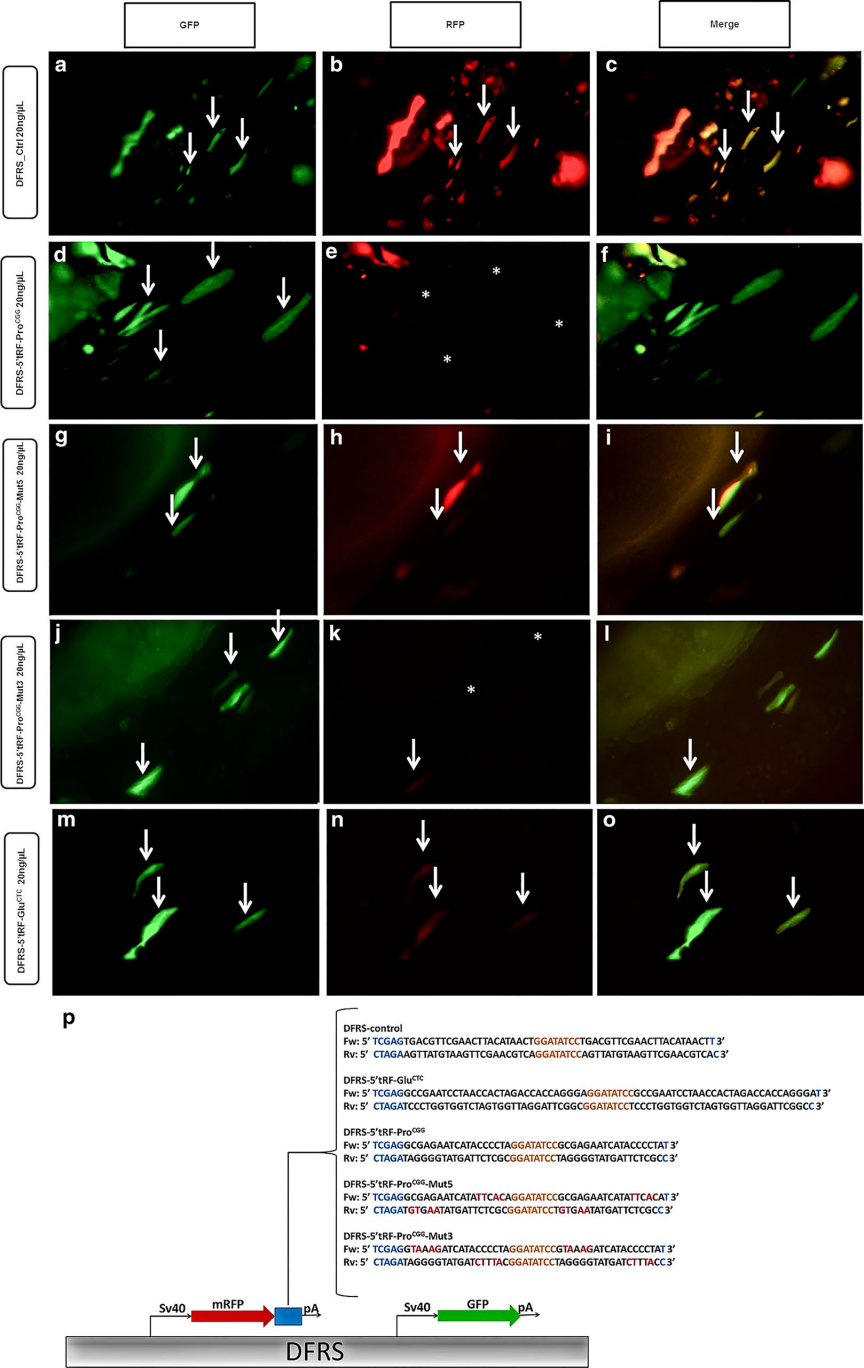
Silencing ability of tRFs using a dual reporter plasmid. Endogenous 5′tRF-Pro^CGG^ has silencing ability. Embryos injected with 20 ng/µL of DFRS control plasmid (**a**, **b**, **c**), show GFP and mRFP signal, while the RFP signal is lost after microinjection of 20 ng/µL DFRS-5′tRF-Pro^CGG^ plasmid (**d**, **e**, **f**). 5′ portion of 5′tRF-Pro^CGG^ is necessary for target silencing, as mutations in the reporter that affect binding at the 5′ end (DFRS-5′tRF-Pro^CGG^_Mut5) result in recovery of RFP fluorescence (**g**, **h**, **i**) when compared to non-mutated reporter (**d**, **e**, **f**). Mutations that affect binding of the 3′ end of the tRF do not affect silencing ability as RFP is repressed (**j**, **k**, **l**), similarly to the non-mutated reporter. There is only a slight decrease in RFP signal after microinjection of 20 ng/µL DFRS-5′tRF-Glu^CTC^ (**m**, **n**, **o**) plasmid, indicating that the endogenous 5′tRF-Glu^CTC^ does not have trans-silencing ability. *Arrows* indicate cells containing both GFP-reporter and mRFP-sensor. *Asterisks* indicate muscle fibers that lost mRFP fluorescence. Orientation of embryos: caudal, *left*; *ventral*, *up* ×20 magnification. **p** DFRS plasmid scheme. The DFRS plasmid bears two fluorescent proteins, namely GFP (*green*) and mRFP (*red*), controlled by SV40 promoters. The mRFP contains a 3′UTR cassette (*blue box*) complementary to the tRF of interest. The sequences inserted in the DFRS to obtain the different reporters (DFRS-control, DFRS-5′tRF-Glu^CTC^, DFRS-5′tRF-Pro^CGG^, DFRS-5′tRF-Pro^CGG^-Mut5 and DFRS-5′tRF-Pro^CGG^-Mut3) are depicted. Restriction sites are shown in *blue*, *Eco*RV cleavage site is shown in *orange* and the mutated nucleotides are highlighted in *red*

A dual fluorescence reporter system (DFRS) consisting of a GFP-Reporter/mRFP-Sensor plasmid was injected into one cell stage zebrafish embryos to evaluate the silencing ability of endogenously available 5′tRF-Glu^CTC^ and 5′tRF-Pro^CGG^. This reporter expresses both RFP and GFP under the control of the same promoter. The RFP contained a 3′UTR cassette complementary to the tRF of interest, which functions as a silencing sensor; the GFP lacks complementary sites functioning as an internal control reporter. While the GFP is always expressed in cells that incorporate the plasmid, the expression of the RFP reporter is repressed if the endogenous tRF of interest has trans-silencing activity. We have observed a decrease in the expression of the RFP signal relative to the control at 24 hpf upon injection of the DFRS-5′tRF-Pro^CGG^ (Fig. 5a–f). The silencing was still observed at 72 hpf (Additional file 2: Figure S2D–F) indicating that the endogenous 5′tRF-Pro^CGG^ binds to its complementary sites and induces silencing. To further confirm this observation, we have engineered mutations in the 5′ and 3′ ends of the RFP sites that were complementary to the 5′tRF-Pro^CGG^ to disrupt binding and silencing (Fig. 5p). We have assumed that any mutation would affect silencing if full complementarity between the tRF and its target was required or that silencing would be affected by specific mutations only if partial complementarity, similar to that used by miRNAs, would be required. We have observed that the mutations that affected the binding of the 5′end of 5′tRF-Pro^CGG^ (corresponding to nucleotides 2, 3, 5 and 6 of the tRF) with the reporter (DFRS-5′tRF-Pro^CGG^-Mut5) derepressed RFP expression, indicating that these sites are essential for endogenous 5′tRF-Pro^CGG^ silencing activity (Fig. 5g–i). Injection of DFRS-5′tRF-Pro^CGG^-Mut3, which affects binding of 5′tRF-Pro^CGG^ 3′end to complementary target sites (corresponding to nucleotides 13, 14, 16 and 17 of the tRF), did not affect RFP expression, indicating that tRF 3′end nucleotides are not essential for target recognition and silencing (Fig. 5j–l).

There was a slight decrease in RFP intensity levels after microinjection of DFRS-5′tRF-Glu^CTC^ relative to GFP, but the RFP signal was not abolished (Fig. 5m–o). Since 5′tRF-Glu^CTC^ levels are low at 24 and 48 hpf we checked for RFP expression after 72 hpf, but no considerable alterations were observed (Additional file 2: Figure S2G–I), indicating that the endogenous 5′tRF-Glu^CTC^ does not silence gene expression as efficiently as 5′tRF-Pro^CGG^. Approximately 50 embryos were microinjected per condition and biological replicate. Three biological replicates were performed for each condition tested.

### tRFs are conserved between zebrafish and humans

**Fig. 6:**
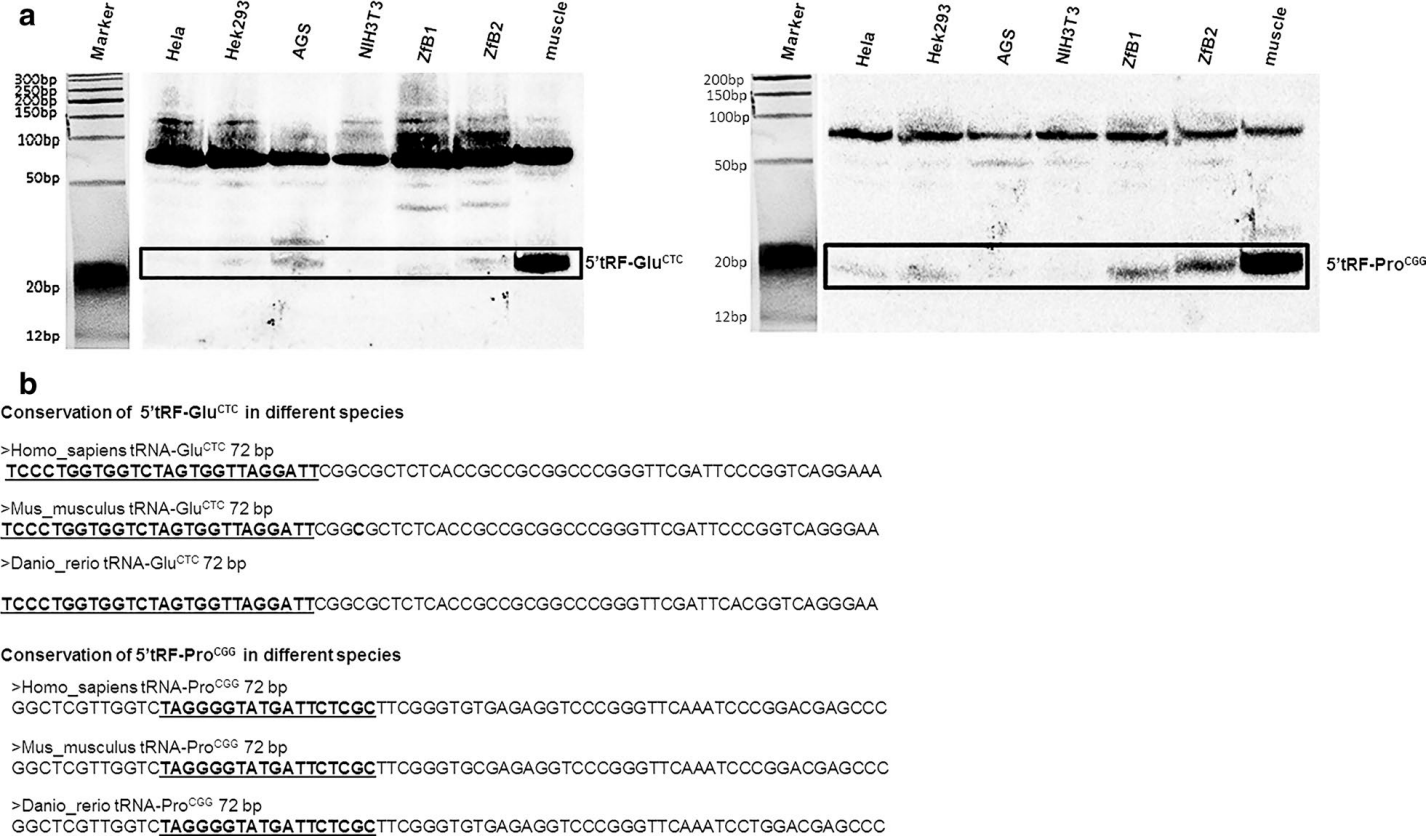
tRFs are conserved among vertebrate species. **a** Northern blots showing the expression of 5′tRF-Glu^CTC^ and 5′tRF-Pro^CGG^ in human cell lines (HeLa, HEK293, AGS), a mouse cell line (NIH3T3) and zebrafish cell lines derived from bone (ZFb1, ZFb2). RNA extracted from adult zebrafish muscle was used as a positive control. 5′tRF-Glu^CTC^ and 5′tRF-Pro^CGG^ were detected in human and zebrafish cell lines, but barely detected in the mouse embryonic fibroblast cell line (NIH3T3). **b** ClustalW alignments of mature tRNA and tRFs showing high conservation in different species, namely human, mouse and zebrafish

Since tRNAs are well conserved across vertebrates, it is plausible that the same is true for tRFs. To validate the conservation hypothesis, total RNA was extracted from different zebrafish, human and mouse cell lines and analyzed by northern blots. ZFb1 and ZFb2 cells derived from jaw, vertebra and branquial arch tissues of zebrafish, HeLa cells derived from human epithelial cervical cancer, HEK293 cells derived from human embryonic kidney, AGS cells derived from human stomach adenocarcinoma and mouse NIH3T3 cells derived from mouse embryonic fibroblasts, were analyzed. RNA from zebrafish bone/muscle was used as a positive control, as it corresponds to a tissue where these tRFs are abundant, as shown previously (Fig. 2). 5′tRF-Glu^CTC^ and 5′tRF-Pro^CGG^ were detected in both human and zebrafish cell lines, and to less extent in mouse NIH3T3 cells (Fig. 6). Both 5′tRF-Glu^CTC^ and 5′tRF-Pro^CGG^ were slightly shorter in the Zfb1 cell line, suggesting slightly different processing in different organisms.

### 5′tRF levels are affected during infection and in colorectal cancer

We have also analysed the presence of tRFs in human sequencing datasets deposited in the GEO database. From the different datasets analysed, we were able to identify 5′tRF-Lys^TTT^, 5′tRF-Val^CAC^, 5′tRF-Glu^CTC^, 5′tRF-Pro^CGG^, 3′tRF-Glu^CTC^ and 3′tRF-Pro^AGG^ in 5 of them, namely GSM1293576 [30], GSE33584 [31], GSE29173 [32], GSE22918 [33] and GSE43550. Low number of reads of 5′tRF-Val^CAC^ and 5′tRF-Glu^CTC^ (maximum 185 reads) were identified in serum of old and young individuals (GSM1293576), with no particular age preference (Additional file 3: Table S1). Both tRFs identified were 4 nt longer than the original zebrafish tRFs, probably indicating variation in processing of these molecules in human serum.

3′tRF-Pro^AGG^ was the only zebrafish tRF identified in breast tissue (normal and tumour tissue)—GSE29173—, but no significant change in read numbers was found between samples (Additional file 3: Table S1). Only 5′tRF-Glu^CTC^ reads were found in the nucleus and in the cytoplasm of 5-8F cells—a nasopharyngeal carcinoma cell line (GSE22918). Reads corresponding to other tRFs, namely 5′tRF-Lys^TTT^, 5′tRF-Pro^CGG^, 3′tRF-Glu^CTC^ and 3′tRF-Pro^AGG^ were only found in the cytoplasm of 5-8F cells, indicating that most tRFs are present in the cytoplasm rather than in the nucleus (Additional file 3: Table S1). This is not surprising as mature cytoplasmic tRNAs are the precursors of most tRFs, namely 3′ and 5′tRFs [34]. The fact that 5′tRF-Glu^CTC^ was found in the nucleus indicates that tRFs migrate to this organelle, similarly to what has already been found for other sncRNAs, namely miRNAs [35, 36].

**Fig. 7:**
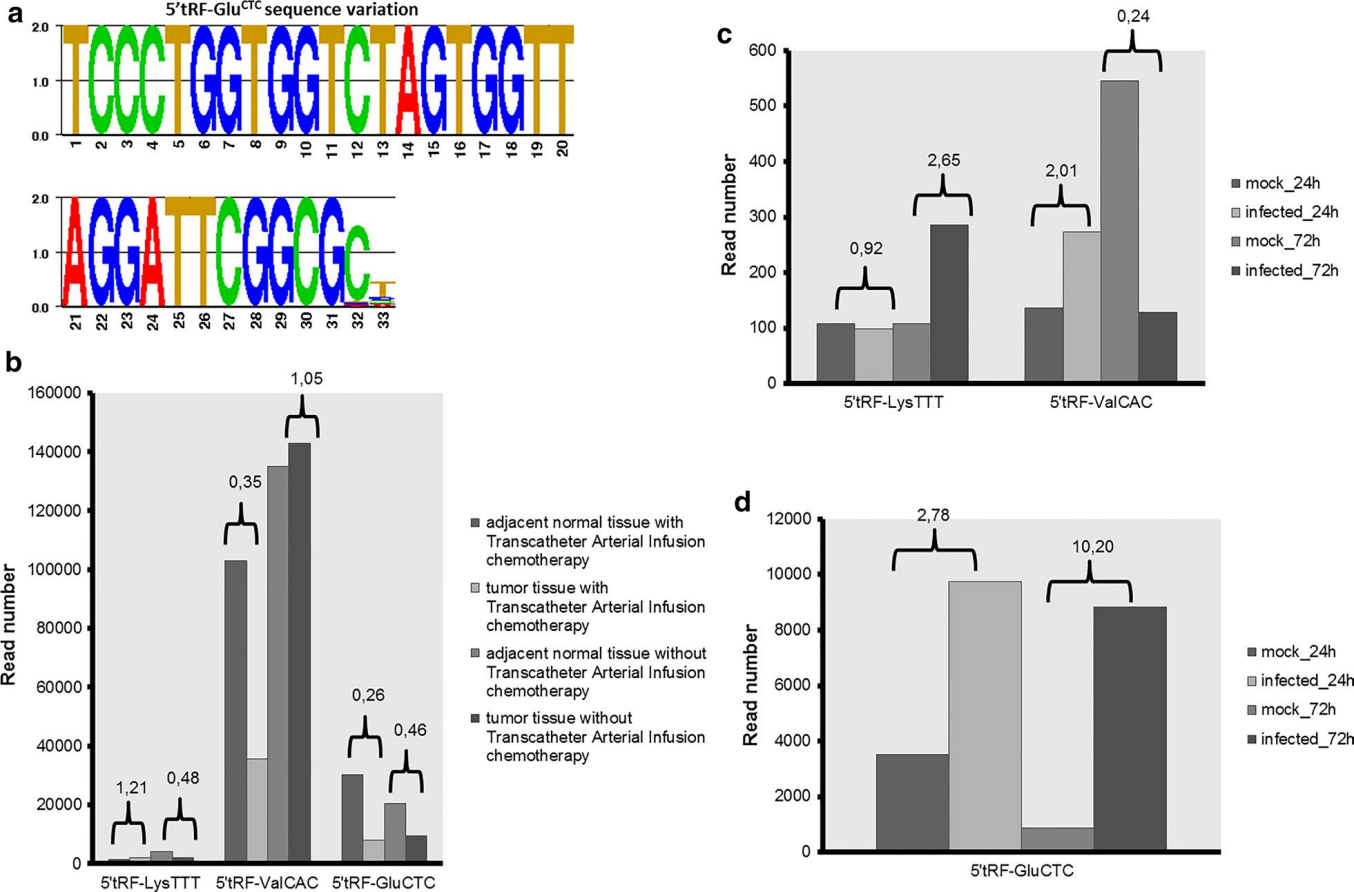
Analysis of zebrafish tRFs in human samples. **a** Sequence variation of 5′tRF-Glu^CTC^ in colorectal cancer datasets. Sequence logo was generated by GENIO/logo. **b**, **c**, **d** *Graphs* depicting the read numbers for 5′tRF-Lys^TTT^, 5′tRF-Val^CAC^ and 5′tRF-Glu^CTC^ in normal tissues vs sample (colorectal cancer **b**; infection **c**, **d**). Ratios between condition and control are *highlighted*

Analysis of the sequencing data from colorectal tumour samples (GSE43550) revealed the presence of 5′tRF-Lys^TTT^, 5′tRF-Val^CAC^, 5′tRF-Glu^CTC^ and 5′tRF-Pro^CGG^, both in adjacent normal and in the tumour tissue. These tRFs, in particular 5′tRF-Glu^CTC^ showed sequence variations at the 3′ end, indicating that the cleavage events are not precise even in the same tissue, similarly to miRNAs [37] (Fig. 7a; Additional file 3: Table S1). 5′tRF-Pro^CGG^ was only detected in tissues (normal and tumour) without transcatheter arterial infusion chemotherapy. 5′tRF-Lys^TTT^ reads decreased in tumour samples without arterial infusion chemotherapy, but no significant difference in read number was detected between normal and tumour samples with treatment. A slight decrease (~7000 reads difference) was detected for 5′tRF-Val^CAC^ in tumour tissue without transcatheter arterial infusion chemotherapy relative to the corresponding normal tissue. The expression of this tRF seemed to be affected by transcatheter arterial infusion chemotherapy as the total number of reads decreased from ~100,000 (normal tissue with transcatheter arterial infusion) to ~35,000 (tumour tissue with transcatheter arterial infusion). 5′tRF-Glu^CTC^ was also down regulated in tumour tissues, independently of transcatheter arterial infusion chemotherapy. The total number of 5′tRF-Glu^CTC^ reads decreased from ~30,000 (normal adjacent tissue with infusion chemotherapy) to ~8000 (tumour tissue with infusion chemotherapy) and decreased to about half that number in tumour tissue without infusion chemotherapy compared to its corresponding normal tissue (Fig. 7b). The decrease in read number was always observed for all the isoforms (different sequences with 3′end variations) and some of them were absent in tumour samples. This data show that 5′tRF-Val^CAC^ and especially 5′tRF-Glu^CTC^ may have a role in colorectal cancer and have potential to be used as cancer biomarkers.

5′tRF-Lys^TTT^, 5′tRF-Val^CAC^ and 5′tRF-Glu^CTC^ were identified in 24 and 72 h non-infected and cytomegalovirus infected primary human fibroblasts (GSE33584). The three identified tRFs were always 2 nt longer when compared to the original zebrafish tRF. Read numbers were relatively low for 5′tRF-Lys^TTT^ and 5′tRF-Val^CAC^, but still 5′tRF-Lys^TTT^ reads were fourfold higher in infected cells 72 h post-infection while 5′tRF-Val^CAC^ reads were fourfold lower in 72 h post-infected cells. The number of 5′tRF-Glu^CTC^ reads was ~threefold higher 24 h post-infection and ~tenfold higher 72 h post-infection, indicating a role for this tRF during infection (Additional file 3: Table S1; Fig. 7c, d). This particular tRF and 5′tRF-Lys^TTT^, among others, were up-regulated upon respiratory syncytial virus infection in airway epithelial cells (A549 cells) and were implicated in RSV replication [14]. In this case, 5′tRF-Glu^CTC^ was only 1 nt longer than the zebrafish 5′tRF-Glu^CTC^ and, consequently, 1 nt shorter than the 5′tRF-Glu^CTC^ detected in human fibroblasts. Since 5′tRF-Glu^CTC^ variability was found in different cell lines it is likely that the 3′end variability is tissue specific and reflect tissue specific variation in processing. 5′tRF-Lys^TTT^ had no variation in A549 cells relative to the zebrafish tRF and 5′tRF-Glu^CTC^ was abundant after rickettsia infection in mice and exhibited no sequence variation when compared to the equivalent zebrafish tRF [38].

## Discussion

### tRFs are highly expressed in adult zebrafish

In this study we have identified 10 zebrafish tRFs—4 belonging to the tRF-5 series and 6 belonging to the tRF-3 series—distributed throughout development and adult tissues. Since our deep sequencing experiment was designed to identify miRNAs with a NGS read length cutoff between 15 and 30 nt, it is likely that longer tRFs that are generally induced by nutritional deprivation and stress conditions were missed [20, 22, 39]. Studies are needed to clarify this question.

Several tRFs were previously identified in zebrafish early developmental stages, up to 24 hpf [37]. Sequencing of sncRNAs in different developmental stages, namely 256-cell, sphere, shield and 24 hpf showed that tRFs are expressed at low level during early development, but there is a slight enrichment at the 256 cell stage and at 24 hpf [37]. Our analysis started at 24 hpf, when most tRFs are not expressed and progressed to zebrafish tissues where tRFs are abundantly expressed. Most tRFs detected in previous studies [37] were not identified by us, probably due to the higher sequencing coverage used [26, 37]. Alternatively, those tRFs are present during early development and were missed because we did not analyze the early developmental stages. It is also possible that those tRFs are expressed at exceptionally low levels and were missed by our NGS experiment, as we used a chip that retrieves a maximum of 100,000 reads. Nevertheless, 3′tRF-Pro^AGG^ was present in 256-cell, sphere stages and 24 hpf. 5′tRF-Pro^CGG^ was also present at 24 hpf [35]. Furthermore, analysis of zebrafish sequencing datasets available in GEO showed that these two tRFs are present in endothelial cells of 24 hpf embryos [40].

We have found that the cellular concentration of tRFs is not correlated with the abundance of the corresponding tRNAs, which is an indication that these molecules are not products of random tRNA degradation, as previously shown [10]. However, in some cases, namely 5′tRF-Pro^CGG^, an alternative band of approximately 30 nt was detected in adult ZF tissues, indicating that tRF processing may differ across different tissues or developmental stages and thus, different regulation mechanisms of tRF generation may exist and should be further explored in the future.

Northern blot analysis of the tRFs identified by NGS showed that they are highly expressed in mature tissues, such as bone and skin. One of the most striking results was obtained for 5′tRF-Pro^CGG^ whose higher abundance in the skin and bone/muscle relative to the corresponding mature tRNA may suggest a role in these particular tissues that needs to be further explored. Its in vitro cleavage by Dicer supports this hypothesis as it allows for incorporation into the RNAi pathway [10, 17, 18], but in vivo cleavage should be tested in the future with zebrafish dicer mutants [41]. How Dicer recognizes these tRNAs still needs to be clarified. One possibility is that the pre-tRNA transcripts may have alternative folds with long hairpins that are recognized and cleaved by Dicer [13, 19]. Coincidently, part of the mature tRNAs that originate 5′tRF-Glu^CTC^ and 5′tRF-Pro^CGG^ can form long hairpins recognizable by Dicer. It will be interesting to experimentally validate these folding predictions to clarify the role of tRNA folding in the biogenesis of tRFs. Moreover, the folding of tRNAs into alternative secondary and tertiary structures may also explain the existence of tRNA gene duplications in the genomes of vertebrates and the lack of correlation between the abundance of mature tRNAs and their respective tRFs.

### Zebrafish tRFs are conserved in vertebrates

We have shown by northern blot analysis that 5′tRF-Glu^CTC^ and 5′tRF-Pro^CGG^ are conserved in vertebrates, namely in mouse and in human, which is supported by published studies. Indeed, the 5′tRF-Glu^CTC^ is described in a human fetus hepatic tissue [20], a human monocytic cell line (THP-1) [13] and in adenocarcinomic human alveolar basal epithelial cells (A549) [14]. The tRNA^Glu^ fragment cloned from human fetus hepatic tissue is 3 nt longer than the zebrafish 5′tRF-Glu^CTC^, whereas the 5′tRF-Glu^CTC^ identified in THP-1 cells and A549 is 2 nucleotides and 1 nucleotide shorter, respectively, suggesting that the 3′-end of this fragment is heterogeneous, similarly to miRNAs. This may be due to mismatches between the retrieved sequences and the genomic loci, but the cleavage position may also vary with the cell type or tissue [4, 37] due to differential processing [5]. Besides, our analysis of publically available NGS datasets identified several of the zebrafish tRFs described in our study, namely 5′tRF-Lys^TTT^, 5′tRF-Val^CAC^, 5′tRF-Glu^CTC^, 5′tRF-Pro^CGG^, 3′tRF-Glu^CTC^ and 3′tRF-Pro^AGG^. Taken together, our data confirms the conservation of tRFs in vertebrates, suggests conserved tRF processing pathways and tissue specific expression of the tRFs.

### 5′tRF-Pro^CGG^ has silencing ability

Dicer-dependent tRFs share some features of the RNAi pathway and may represent a class of sncRNA molecules with specific functions, including gene silencing. Silencing has been demonstrated with standard reporter assays for a Dicer-dependent tRF [18] and a recent study showed that a Dicer dependent 3′tRF can repress expression of a set of endogenous genes, including RPA1, by binding to target sites on the mRNA 3′UTR, similarly to miRNAs [17]. Our study shows that 5′tRF-Pro^CGG^ functions in the silencing pathway, as the endogenous 5′tRF-Pro^CGG^ is able to induce silencing of a RFP reporter by binding to complementary target sites present on its 3′UTR. This miRNA-like feature was further confirmed by loss of silencing of a target gene mutated on the seed region of the tRF (between nucleotides 2 and 8). Since this tRF is highly expressed in bone and skin it will be interesting to validate putative gene targets to understand its functional role. We have predicted computationally putative targets for this tRF, as described before [26], by maintaining the seed match between nucleotides 2 and 7, and allowing up to 6 mismatches in the remaining sequence. Some of the predicted target genes are involved in embryonic patterning, cartilage and skeletal development, namely sec23b and myst3, which is consistent with the expression pattern of 5′tRF-Pro^CGG^ (Additional file 4: Table S2).

Endogenous 5′tRF-Glu^CTC^ did not silence efficiently the reporter used in this study, despite its apparent Dicer dependent production in vitro. This could be explained by low levels of endogenous 5′tRF-Glu^CTC^ at 24 hpf, however even at 72 hpf there was no obvious RFP silencing, meaning that this fragment does not have miRNA-like trans-silencing ability. Recently 5′tRF-Glu^CTC^ was identified in A549 cells after respiratory syncytial virus infection and the authors demonstrated that silencing of a reporter occurred through a mechanism distinct from the miRNA/siRNA pathway [14]. The silencing mechanism was not investigated, but other studies also showed that 5′tRFs can inhibit translation by alternative mechanisms. For example, Sobala and colleagues demonstrated that translational inhibition by 5′tRFs is dependent on a conserved dinucleotide (GG) motif present at position 19 and does not need complementary target sites in the mRNA [16]. These authors also found that endogenous 5′tRFs were not able to silence reporters transfected into cells and that silencing was only observed when a duplex mimic of those tRFs was co-transfected, which is not surprising as the synthetic duplex functions as a siRNA and induces miRNA-like-silencing. General inhibition of translation was achieved when different synthetic 5′tRFs bearing the GG dinucleotide, not in the duplex form, were transfected into cells [16]. A synthetic tRF similar to 5′tRF-Glu^CTC^ was the only 5′tRF that did not exhibit silencing ability, despite the GG motif [16].

### tRFs are differentially expressed during infection and in cancer

5′tRF-Glu^CTC^ is up-regulated after respiratory syncytial virus [14] and cytomegalovirus infection [31] and is down regulated in colon rectal cancer, as shown in the results section. Other tRFs are down regulated in cancer. For example, a 3′tRF (also called CU1276) is down-regulated in B cell lymphoma, similarly to 5′tRF-Val^CAC^, 5′tRF-Lys^TTT^ and 5′tRF-Glu^CTC^ in colorectal cancer [17]. These data show a trend for down regulation of tRFs in cancer and up regulation during infection, but further validation experiments are needed to clarify whether these molecules can be used as disease biomarkers. For example, it would be interesting to analyze the tRF profile in different cancers and under different types of infections and also in different populations and experimentally validate the sequencing data by northern blot and qPCR techniques.

## Conclusions

Our data show that tRFs are conserved in vertebrates and confirms that tRFs are expressed in a cell and tissue specific manner. For example, in zebrafish the identified tRFs are mostly expressed in muscle, bone and skin and less expressed in the brain and during development, which is probably related to its biological function. Moreover, our data shows that 5′tRF-Pro^CGG^ is more expressed in these tissues than its corresponding mature tRNA and that it can play a role in gene expression regulation as it exhibits silencing ability. It is now important to experimentally validate the biological targets of these molecules and determine if those targets are correlated with its high expression. Our data also shows that besides being conserved in vertebrates, tRF expression is affected in specific disease conditions, namely infection and cancer. The differential expression of tRFs in these conditions indicates that tRFs have the potential to be used as disease biomarkers. It would be interesting to analyze the available NGS datasets of human diseases to identify all the tRFs present in specific samples and identify situations where these molecules are deregulated. Their similarity with miRNAs may allow them to recruit the miRNA machinery, but they may have their own machinery or cooperate with other sncRNAs to control important biological processes.

## Methods

### Zebrafish husbandry

Wild type AB zebrafish strain was obtained from the fish facility at the Department of Biology, University of Aveiro and maintained at 28 °C on a 14 h-light/10 h-dark cycle. Zebrafish maintenance followed the Portuguese law for animal experimentation (Regulatory Guideline no 113/2013, 7th August, 2013) and the experiments were approved by the National Food and Veterinary Authority (DGAV) in Portugal and by the committee for animal experimentation and well-being of the Biology Department, University of Aveiro.

### Computational analysis of sequencing reads

Base calling and quality trimming of sequence reads was carried out as described before [42]. For the identification of tRFs, reads that did not match miRNAs were aligned against a small RNA database extracted from Biomart/Ensembl. Up to two mismatches were allowed in alignments to ensure that sequences with sequencing errors or post-transcriptional modifications, which could produce reverse transcription errors during cDNA library construction, were not discarded. tRFs identified were then aligned against their mature tRNA to verify if the sequences were not randomly distributed through the mature sequence.

### RNA analysis

Total RNA was extracted from zebrafish embryos, adult tissues and cultured cells with TRIZOL^®^ (Invitrogen). Twenty micrograms of total RNA was fractionated on 10 % denaturing polyacrylamide (PAA) gels. RNA was then transferred during 30 min to Hybond-N membranes (GE Healthcare) using a semidry transfer system and was UV-crosslinked. Antisense oligonucleotides complementary to predicted tRF candidates were radio labeled with [^32^P]-ATP and T4 polynucleotide kinase (Takara) and were used as hybridization probes. Membranes were pre-hybridized for 4 h in pre-hybridization/hybridization buffer containing 5× Denhardt’s solution, 1 % SDS and 6.6× SSPE at 64 °C (5′tRF-Lys^TTT^ and 5′tRF-Glu^CTC^), 40 °C (5′tRF-Pro^CGG^), 57 °C (3′tRF-Pro^AGG^ and 3′tRF-Ala^AGC^ probe) and 56 °C (U6). Membranes were stripped twice and probed with a maximum of three probes. For the stripping, membranes were incubated with a solution of 50 % formamide and 2xSSPE at 65 °C for 1 h, followed by 15 min incubation with TE buffer to neutralize the membrane. [^32^P]-ATP labeled probes were added to the hybridization chamber and incubated with the membranes overnight at the mentioned temperatures. Membranes were then washed twice with washing solution, containing 2xSSPE and 0.1 % SDS at room temperature and twice with washing solution at 57 °C for 3 min, and were exposed to a phosphor screen (Biorad^®^) overnight and scanned using a Molecular Imager^®^ FX (Biorad), equipped with Quantity One FX software.

Probes:

5′tRF-Lys^TTT^: 5′-CTGATGCTCTACCGACTGAGCTATCCGGGC-3′

5′tRF-Glu^CTC^: 5′-GCCGAATCCTAACCACTAGACCACCAGGGA-3′

5′tRF-Pro^CGG^: 5′-GCGAGAATCATACCCCTA-3′

3′tRF-Pro^AGG^: 5′-TGGGGGCTCGTCCGGGA-3′

3′tRF-Ala^AGC^: 5′-GGGCATCGATCCCGCTACCTCT-3′

U6: 5′-AATATGGAACGCTTCACGAATTTGCGTGTC-3′

### Angiogenin and dicer in vitro assays

For the Angiogenin cleavage assay, 20 µg of total RNA extracted from 72 h post fertilization (hpf) zebrafish embryos were incubated with 1 µM of the enzyme in PBS + 0.1 % BSA for 10 min, 30 min, 1 and 2 h at 37 °C. The cleaved products were recovered using phenol/chlorophorm extraction followed by ethanol precipitation. Approximately 20 µg of each sample were fractionated on 10 % PAA gels and transferred onto Hybond-N membranes for northern blot analysis as described previously.

For the Dicer assay, recombinant human Dicer enzyme kit from Genlantis was used according to the manufacturer’s instructions with minor changes. Briefly, 20 µg of total RNA extracted from 72 hpf zebrafish embryos were incubated with 2 U of recombinant Dicer in 20 µL reactions, for 30 min, 1, 2, 4 and 6 h. Reactions were stopped with Dicer Stop Solution and were electrophoresed on 10 % PAA gels and transferred onto Hybond-N membranes for northern blot analysis, as described previously.

### Dual reporter assay

To test tRFs silencing ability, a dual fluorescence reporter system (DFRS)—pDSV2-eGFP-mRFP was used. This reporter bears two fluorescent proteins, GFP and RFP, controlled by the same promoter. GFP identifies the tissues expressing the plasmid and the RFP, which contains a 3′UTR cassette complementary to the tRF of interest, functions as a silencing sensor. DFRS plasmids were obtained from DR. Wieland Huttner [43] and slightly modified. Briefly, primers containing multiple cloning sites (*Xho*I-*SaI*I_*Nhe*I), as described in [44] were annealed and inserted into *ECO*RI-*Not*I sites of the DFRS plasmid. Next, two different DFRS plasmids containing complementary sites for 5′tRF-Glu^CTC^ and 5′tRF-Pro^CGG^ and a control DFRS were generated. DFRS-5′tRF-Glu^CTC^, DFRS-5′tRF-Pro^CGG^ and DFRS-control were each cloned into *Xho*I-*Nhe*I site by annealing nucleotides A–B, C–D and E–F, respectively.

To study the 5′tRF-Pro^CGG^ functional domains DFRS-5′tRF-Pro^CGG^ bearing mutations on the 5′ and on the 3′ end were generated. 5′ end mutations of DFRS-5′tRF-Pro^CGG^ were obtained after annealing primers G–H. 3′ end mutations were obtained after annealing primers I–J. Mutated sequences were cloned into *Xho*I-*Nhe*I site.

Primers (written in direction 5′–3′):

A-TCGAGGCCGAATCCTAACCACTAGACCACCAGGGAGGATATCCGCCGAATCCTAACCACTAGACCACCAGGGAT

B-CTAGATCCCTGGTGGTCTAGTGGTTAGGATTCGGCGGATATCCTCCCTGGTGGTCTAGTGGTTAGGATTCGGCC

C-TCGAGGCGAGAATCATACCCCTAGGATATCCGCGAGAATCATACCCCTAT

D-CTAGATAGGGGTATGATTCTCGCGGATATCCTAGGGGTATGATTCTCGCC

E-TCGAGTGACGTTCGAACTTACATAACTGGATATCCTGACGTTCGAACTTACATAACTT

F-CTAGAAGTTATGTAAGTTCGAACGTCAGGATATCCAGTTATGTAAGTTCGAACGTCAC

G-TCGAGGCGAGAATCATATTCACAGGATATCCGCGAGAATCATATTCACAT

H-CTAGATGTGAATATGATTCTCGCGGATATCCTGTGAATATGATTCTCGCC

I-TCGAGGTAAAGATCATACCCCTAGGATATCCGTAAAGATCATACCCCTAT

J-CTAGATAGGGGTATGATCTTTACGGATATCCTAGGGGTATGATCTTTACC

### Zebrafish embryos microinjection

One cell zebrafish eggs were microinjected with ~1000 pL of a solution containing 20 ng/µL of the reporter plasmid, phenol red and 0.9 M KCl. Embryos were kept at 28 °C and analyzed at 24 and 72 hpf under an epifluorescence microscope Imager.Z1 (Zeiss), a GFP and mRFP filter, AxioCam HRm camera (Zeiss) and AxioVision software (Zeiss). Approximately 50 embryos were used per replica and per condition. Three biological replicates were performed.

### Target prediction

The 3′UTR sequences of zebrafish mRNAs were extracted from Biomart (http://www.biomart.org) and blasted against the antisense 5′tRF-Pro^CGG^ sequence. Sequences with perfect seed match between nucleotides 2 and 7, and no more than six mismatches in the remaining sequence were retained for further analysis. Targets were considered positive whenever RNAhybrid confirmed them thermodynamically. Targets were discarded when RNAhybrid did not retrieve the targets obtained in the first approach.

### Computational analysis of publicly available sequencing datasets

Publicly available NGS sequencing datasets deposited in GEO were analyzed to identify the tRFs uncovered in zebrafish. Briefly, datasets were downloaded from GEO and a blast search for each tRF was performed. Lists of identified tRFs and respective number of reads were generated for each sample. Only sequences with more than 50 reads were kept for further analysis. All data used corresponded to the processed sequence along with the cloning frequency that was available in all datasets used. Normalized data against the total number of reads corresponding to sncRNAs for each sample was used to compare the relative levels of tRFs in different samples in the same experiment (sample vs control).

## Availability of supporting data

The datasets supporting the results of this article have been deposited in NCBI’s Gene Expression Omnibus [45] and are accessible through GEO Series accession number GSE66718 (http://www.ncbi.nlm.nih.gov/geo/query/acc.cgi?&acc=GSE66718).

## Additional files

10.1186/s12867-015-0050-8 Sequencing data analysis pipeline. Pipeline describing the protocol used for identifying miRNAs and other small non-coding RNAs present in pyrosequencing datasets. All reads that were not considered as being miRNAs were blasted against other small RNAs and were identified. Potential tRFs were catalogued.
10.1186/s12867-015-0050-8 Sensor plasmid at 72 hpf. At 72 hpf GFP and RFP expression is equivalent after the injection of the control reporter (A, B, C). Silencing by endogenous 5′tRF-Pro^CGG^ is still present at 72hpf (D, E, F), as RFP expression is repressed. Endogenous 5′tRF-Glu^CTC^ does not efficiently silence putative targets even at 72hpf, as no alterations in RFP expression is observed when compared to GFP expression (G, H, I). Arrows indicate cells containing both GFP-reporter and mRFP-sensor. Asterisks indicate muscle fibers that lost mRFP fluorescence. Orientation of embryos: caudal, left; ventral, up. 20× magnification.
10.1186/s12867-015-0050-8 Sequencing data relative to zebrafish tRFs found in publicly available GEO datasets. For each experiment the sequences of conserved tRFs identified and corresponding number of reads are displayed. ID corresponds to the tRF identification; “Query” corresponds to the zebrafish tRF that was used as a template to search for similar molecules in the datasets; “Sequence” corresponds to the sequence found in the datasets that is related to the “query”; “Count” gives the total number of reads retrieved for each “sequence”; “Source tag” is the GEO dataset sample ID number; “description” depicts the type of sample where the tRF was found.
10.1186/s12867-015-0050-8 Target predictions for 5′tRF-Pro^CGG^. Seed match between nucleotides 2 and 7 were maintained, and up to 6 mismatches in the remaining sequence were allowed. Two of the target genes predicted play roles in embryonic patterning, cartilage and skeletal development, namely Sec23b and Myst3, which correlates with the expression pattern of 5′tRF-Pro^CGG^ (high expression in bone).
