# Supplementary figures and images for: Conserved and highly expressed tRNA derived fragments in zebrafish
Source: BMC Mol Biol. 2015 Dec 22;16:22. doi: 10.1186/s12867-015-0050-8 (PMC4688932; doi:10.1186/s12867-015-0050-8)

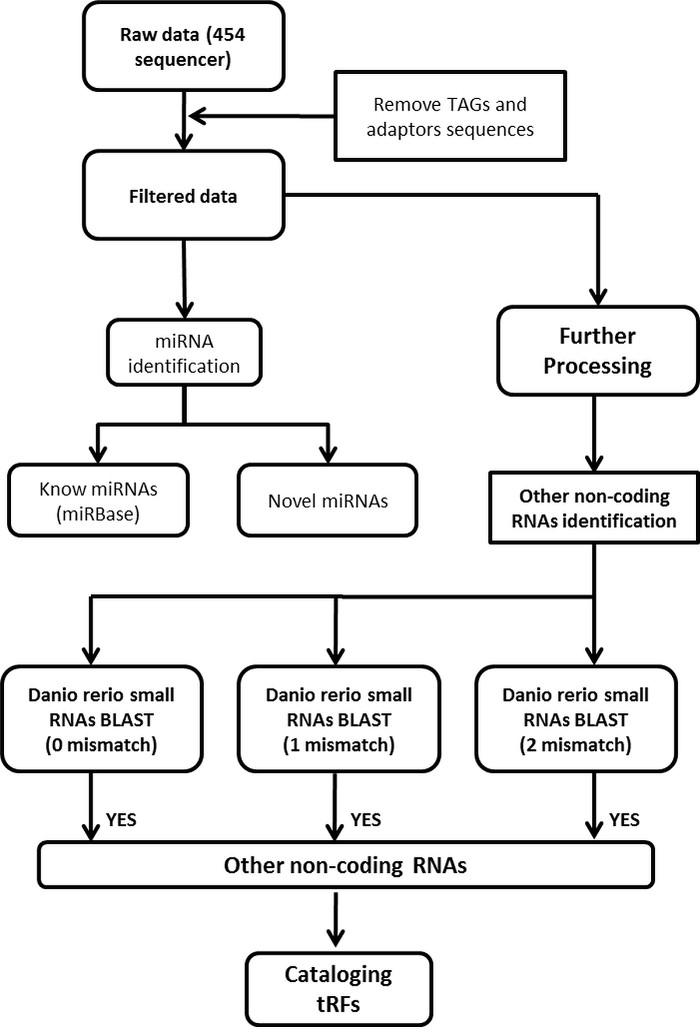

Supplement: Supplementary file 1 — 10.1186/s12867-015-0050-8 Sequencing data analysis pipeline. Pipeline describing the protocol used for identifying miRNAs and other small non-coding RNAs present in pyrosequencing datasets. All reads that were not considered as being miRNAs were blasted against other small RNAs and were identified. Potential tRFs were catalogued. [file 12867_2015_50_MOESM1_ESM.tif]

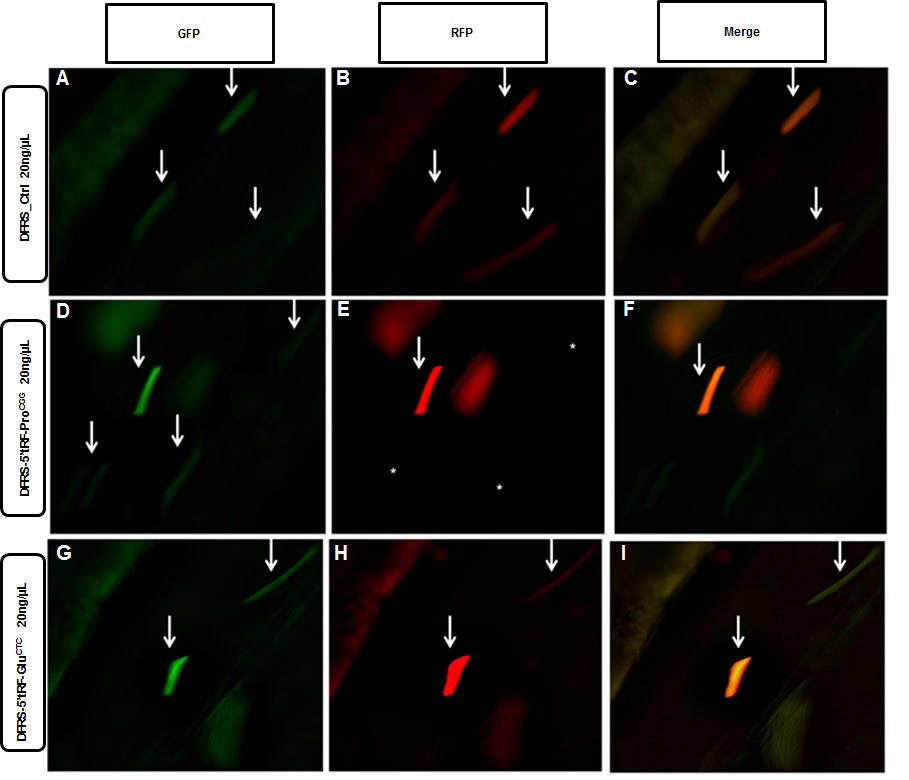

Supplement: Supplementary file 2 — 10.1186/s12867-015-0050-8 Sensor plasmid at 72 hpf. At 72 hpf GFP and RFP expression is equivalent after the injection of the control reporter (A, B, C). Silencing by endogenous 5′tRF-ProCGG is still present at 72hpf (D, E, F), as RFP expression is repressed. Endogenous 5′tRF-GluCTC does not efficiently silence putative targets even at 72hpf, as no alterations in RFP expression is observed when compared to GFP expression (G, H, I). Arrows indicate cells containing both GFP-reporter and mRFP-sensor. Asterisks indicate muscle fibers that lost mRFP fluorescence. Orientation of embryos: caudal, left; ventral, up. 20× magnification. [file 12867_2015_50_MOESM2_ESM.tif]
