# Supplementary material for: Conserved and highly expressed tRNA derived fragments in zebrafish
Source: BMC Mol Biol. 2015 Dec 22;16:22. doi: 10.1186/s12867-015-0050-8 (PMC4688932; doi:10.1186/s12867-015-0050-8)
Supplement: Supplementary file 4 — 10.1186/s12867-015-0050-8 Target predictions for 5′tRF-ProCGG. Seed match between nucleotides 2 and 7 were maintained, and up to 6 mismatches in the remaining sequence were allowed. Two of the target genes predicted play roles in embryonic patterning, cartilage and skeletal development, namely Sec23b and Myst3, which correlates with the expression pattern of 5′tRF-ProCGG (high expression in bone). [file 12867_2015_50_MOESM4_ESM.pdf]

| **Target gene** | | **Free energy** | **Go Function** | **Go Process** |
|---|---|---|---|---|
| *Sec23b* | **target 5' A UUU G G 3'**<br>**GUG GAAU AUACCCCU**<br>**CGC CUUA UAUGGGGA**<br>**tRF 3' U G 5'** | -25,3 | binding: protein | developmental process: neurocranium morphogenesis; cartilage development |
| *Ptpra* | **target 5' A A CUC U 3'**<br>**GC GG AAUCAUACCCCU**<br>**CG UC UUAGUAUGGGGA**<br>**tRF 3' C 5'** | -24,6 | catalytic activity | cellular process: metabolic |
| *Myst3* | **target 5' A U A A 3'**<br>**GC GAGAG CGUACCCC**<br>**CG CUCUU GUAUGGGG**<br>**tRF 3' A A 5'** | -26,7 | binding: DNA | developmental process: embryonic patterning; cartilage and skeletal development |
| *Parva* | **target 5' U U UU A 3'**<br>**GC G AAUUAUACCCCU**<br>**CG C UUAGUAUGGGGA**<br>**tRF 3' UC 5'** | -23,8 | unknown | unknown |
| *Exoc6* | **target 5' A CA G 3'**<br>**GUGAGG AUACCUU**<br>**CGCUCU UAUGGGG**<br>**tRF 3' UAG A 5'** | -19,2 | unknown | cellular process: exocytosis |
| *Pgm1* | **target 5' C UU A 3'**<br>**G UCAUACCCC**<br>**UC AGUAUGGGG**<br>**tRF 3‘ CGC UU A 5'** | -23,1 | catalytic activity | Cellular process: metabolic |
